# Residual Stress Estimates from Multi-cut Opening Angles of the Left Ventricle

**DOI:** 10.1007/s13239-020-00467-x

**Published:** 2020-06-15

**Authors:** Xin Zhuan, Xiaoyu Luo

**Affiliations:** School of Mathematics and Statistics, University of Glasgow, Glasgow, G12 8SQ UK

**Keywords:** Residual stress, Opening angles, Multiple cuts, Soft tissue, Heterogeneity, Left ventricle

## Abstract

**Purpose:**

Residual stress tensor has an essential influence on the mechanical behaviour of soft tissues and can be particularly useful in evaluating growth and remodelling of the heart and arteries. It is currently unclear if one single radial cut using the opening angle method can accurately estimate the residual stress. In many previous models, it has been assumed that a single radial cut can release the residual stress in a ring of the artery or left ventricle. However, experiments by Omens *et al*. (Biomech Model Mechanobiol 1:267–277, 2003) on mouse hearts, have shown that this is not the case. The aim of this paper is to answer this question using a multiple-cut mathematical model.

**Methods:**

In this work, we have developed models of multiple cuts to estimate the residual stress in the left ventricle and compared with the one-cut model. Both two and four-cut models are considered. Given that the collagen fibres are normally coiled in the absence of loading, we use the isotropic part of the Holzapfel-Ogden strain energy function to model the unloaded myocardium.

**Results:**

The estimated residual hoop stress from our multiple-cut model is around 8 to 9 times greater than that of a single-cut model. Although in principle infinite cuts are required to release the residual stress, we find four cuts seem to be sufficient as the model agrees well with experimental measurements of the myocardial thickness. Indeed, even the two-cut model already gives a reasonable estimate of the maximum residual hoop stress. We show that the results are not significantly different using homogeneous or heterogeneous material models. Finally, we explain that the multiple cuts approach also applies to arteries.

**Conclusion:**

We conclude that both radial and circumferential cuts are required to release the residual stress in the left ventricle; using multiple radial cuts alone is not sufficient. A multiple-cut model gives a marked increase of residual stress in a left ventricle ring compared to that of the commonly used single-cut model.

## Introduction

Living tissues in the heart continuously interact with their bio-environment, reshape and rearrange their constituents under chemical, mechanical or genetic stimuli during their life cycles. In the mature period, the tissues of a healthy heart remain in a homeostatic state. However, heart diseases disrupt this balance, and cause the tissues to grow and remodel. Physiologically, exercise may also induce healthy and reversible growth and remodelling. An important ingredient in evaluating the mechanics involved in the cardiovascular system is knowledge of the solid mechanical properties of the soft tissues involved, including the components of the heart, such as the left ventricle, henceforth abbreviated as LV. A particular aspect is that the tissues of the heart are residually stressed, so that when the external loading is removed, residual stresses remain in the material. However, residual stresses, which are generally assumed to result from growth and remodeling, are imprecisely characterized (experimentally) at present, and how best to include the important effects of residual stresses in cardiovascular applications therefore presents a modelling challenge.

Over the last century,^13^ various hypotheses on the growth and remodelling response to mechanical loading have been put forward, with particular success in arteries. In conventional elasticity theory the existence of a stress-free reference configuration that coincides with the unloaded configuration is normally assumed.^19^ In the present context, however, the unloaded configuration is not stress free, but is residually stressed. The residual stress can be estimated using the so-called *opening angle method*,^2^,^14^ in which an opening angle indicative of the extent of the residual stress can be measured after a single radial cut of an unloaded arterial ring. Using the opened configuration as the reference configuration, the residual stress in a cylindrical artery model can then be estimated.^2^,^26^ This methodology has been extended to multiple cuts by Taber and Humphrey,^27^ and used in a two-layered arterial model by Holzapfel *et al*.^10^

Inclusion of residual stress is important in modelling the mechanics of soft tissues for a number of reasons: (a) in nonlinear elasticity theory the stress state in the reference configuration can have a substantial effect on the subsequent response to loads, and omission of the residual stress can lead to significantly different stress predictions under load^9^,^16^,^24^; (b) in biological tissues, the growth and remodelling process will significantly affect the (residual) stress statement in living tissue^1^,^15^; (c) while the detailed process of local growth is difficult to measure, residual stress, on the other hand, may be estimated from experiments, as demonstrated by the opening angle measurement. Thus, estimates of the residual stress at particular time instants could provide useful information about the growth history of living tissues.

**Figure 1:**
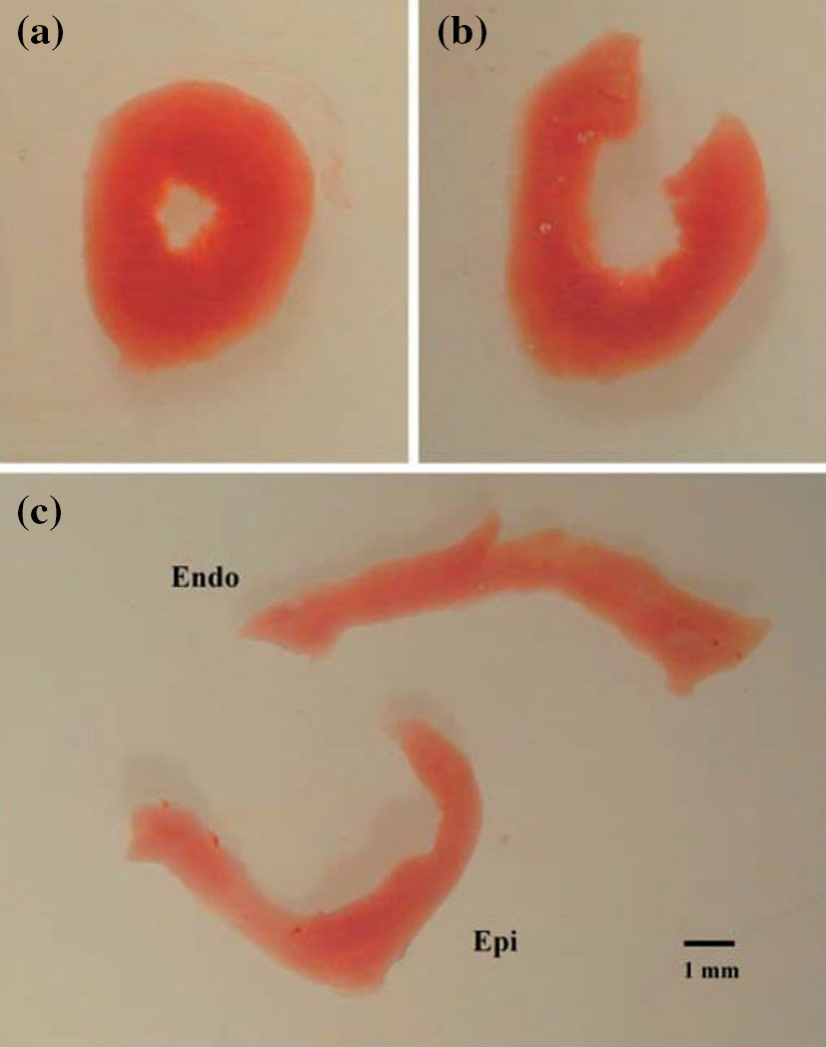
A typical short-axis apical segment of a mouse heart before and after cuts.^21^ The initial intact segment, shown in **a**, was about 2 mm thick. The same segment after a single radial cut and a further circumferential cut is shown in **b** and **c**, respectively. In particular, the endocardial segment has reversed its curvature, in **c**. Note that the definition of the opening angle in Ref. 21 follows that in Chuong and Fung,^2^ which is different from that used in the present paper. Reproduced from^21^ with permission.

However, most work that includes residual stress in complex organs, such as the arteries and heart, assumed that a simple radial cut can release all the residual stresses.^23^,^22^,^7^ However, this assumption is not supported by all experiments. For example, Omens *et al*.^21^ showed that residual stress in a primary mouse heart could be further released by a circumferential cut following the initial radial cut, as illustrated in Fig. 1, They also showed that the opening angles are location-dependent, with greater values at heart apex. This implies that the single-cut opening angle configurations does not correspond to the stress-free configuration, and, as is well known, can only be considered as approximately stress-free. Holzapfel and Ogden^12^ used a three-layer (adventitia, media and intimal) model to study residual stress in the artery, where they found each layer has a different opening angle when cutting open separately. In other words, it is impossible to release all the residual stress from a single radial cut across the three layers. By developing a model to count for the three-layer structure of the artery, the estimated residual stress is much greater than treating the artery as a single-layer model.

Myocardium does not have such a distinct layer structure; however, it is clear from the experiment by Omens *et al*.^21^ that multiple cuts need to be considered when studying residual stress in the heart. Inspired by the finding in Ref. 21 and the three-layer modelling work by Holzapfel and Ogden^12^ on arteries, in this paper, we estimate the residual stress distribution across the wall of an intact mature heart using multiple cuts in a simplified heart model. Unlike the approach in Ref. 12 where the three distinct artery layers are modelled with different material properties, we know that any material property change across the myocardium must be smooth and that the transmural stress distributions are continuous. These considerations are taken into account in the current work.

## Methodology

There is experimental evidence that the collagen fibres are coiled and wavy in their unloaded state in arteries^3^,^5^ and heart.^22^ Similarly to previous studies,^12^,^28^ we assume that collagen fibres are not in tension in the unloaded configuration of the myocardium. Therefore, for modelling the residual stress, we use the isotropic part of the invariant-based constitutive law for the myocardium developed by Holzapfel and Ogden^11^:

1 $$\begin{aligned} \Psi =\frac{a}{2b} \{ \exp [b(I_1-3)]-1\}, \end{aligned}$$

where $I_1=\text{ tr }{\mathbf {C}}=\text{ tr }({\mathbf {F}}^{\mathrm {T}}{\mathbf {F}})$, and *a*, *b* are material constants. The Cauchy stress tensor is then^11^

2 $$\begin{aligned} \varvec{\sigma } =-p {\mathbf {I}}+2\frac{\partial \Psi }{\partial I_1} {\mathbf {B}}, \end{aligned}$$

where ${\mathbf {B}}=\mathbf {FF}^{\mathrm {T}}$ is the left Cauchy–Green deformation tensor.

For simplicity, we model the LV as an incompressible single-layered cylindrical tube. We consider different scenarios based on different numbers of cuts and assume that the stress in the tube in the absence of loads on its curved surfaces can be released by either a single (radial) cut or multiple cuts (a radial cut followed by one or three circumferential cuts). We also assume that all the cut segments retain their cylindrical configurations, each with its own opening angle, i.e. each is a circular cylindrical sector.

### One Cut: Radial

**Figure 2:**
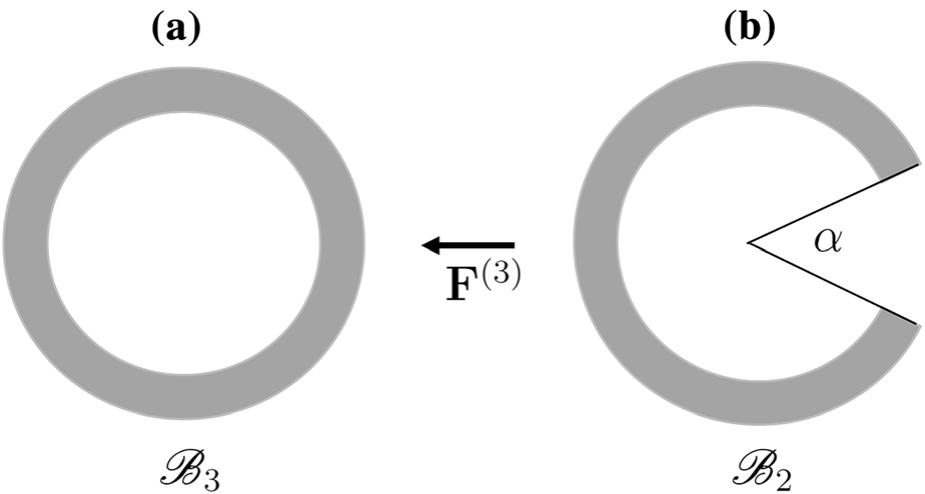
(**a**) Cross-section of a circular cylindrical LV model with no loading on its circular boundaries in configuration ${\mathscr {B}}_3$; **b** Stress-free circular cylindrical sector ${\mathscr {B}}_2$ after a single radial cut from ${\mathscr {B}}_3$. Note that maintenance of ${\mathscr {B}}_3$ as a circular cylindrical configuration requires axial and torsional loads. The deformation gradient from ${\mathscr {B}}_2$ to ${\mathscr {B}}_3$ is denoted ${\mathbf {F}}^{(3)}.$

We take basis vectors $\{ {\mathbf {e}}_r, {\mathbf {e}}_{ \theta }, {\mathbf {e}}_z\}$ to correspond to the local radial, circumferential and longitudinal directions, respectively, in the intact circular cylindrical ring (${\mathscr {B}}_3$). For a single (radial) cut, the opening angle approach has been well described^2^,^10^ for arteries, but is summarised briefly here for completeness. Let the geometry of the right-hand panel in Fig. 2 represent a stress-free configuration ${\mathscr {B}}_2$, which is assumed to be a circular cylindrical sector described by cylindrical polar coordinates $\{R, \Theta , Z \}$ as

3 $$R^{{({\text{i}})}} \le R \le R^{{({\text{o}})}} ,\quad \frac{{\alpha }}{2} \le \Theta \le 2\pi - \frac{{\alpha}}{2},\quad 0 \le Z \le L,$$

where $R^{(\mathrm {i})}$, $R^{(\mathrm {o})}$, and *L* denote the inner and outer radii, and the tube length, respectively, and $\alpha$ is the opening angle. Let $\{{\mathbf {E}}_R,{\mathbf {E}}_\Theta ,{\mathbf {E}}_Z\}$ be the associated cylindrical polar basis vectors in ${\mathscr {B}}_2$.

The (isochoric) deformation from ${\mathscr {B}}_2$ to the intact configuration ${\mathscr {B}}_3$ is then expressed as

4 $$\begin{aligned} {\mathbf {x}}=r {\mathbf {e}}_r+z{\mathbf {e}}_z, \end{aligned}$$

where, by incompressibility,

5 $$\begin{aligned} r= & {} \sqrt{\frac{R^2-{R^{(\mathrm {i})}}^2}{k \lambda _z^{(3)}}+{r^{(\mathrm {i})}}^2}, \quad \nonumber \\ \theta= & {} k (\Theta -\alpha/2), \quad z=\lambda _z^{(3)} Z, \end{aligned}$$

$r^{(\mathrm {i})}$ being the inner radius in the configuration ${\mathscr {B}}_3$, $\lambda _z^{(3)}=l/L$ is the constant axial stretch from ${\mathscr {B}}_2$ to ${\mathscr {B}}_3$, *l* is the cylinder length in ${\mathscr {B}}_3$, and $k=2\pi /(2\pi -\alpha)$ is a measure of the opening angle in ${\mathscr {B}}_2$. The outer radius in ${\mathscr {B}}_3$ is

6 $$\begin{aligned} r^{(\mathrm {o})}=\sqrt{\frac{{R^{(\mathrm {o})}}^2-{R^{(\mathrm {i})}}^2}{k \lambda _z^{(3)}}+{r^{(\mathrm {i})}}^2}. \end{aligned}$$

The corresponding deformation gradient (from ${\mathscr {B}}_2$ to ${\mathscr {B}}_3$), denoted ${\mathbf {F}}^{(3)}$, is given by

7 $$\begin{aligned} {\mathbf {F}}^{(3)}=\lambda _1^{(3)} {\mathbf {e}}_r\otimes {\mathbf {E}}_{R} +\lambda _2^{(3)}{\mathbf {e}}_{\theta }\otimes {\mathbf {E}}_{\Theta } + \lambda _z^{(3)} {\mathbf {e}}_z \otimes {\mathbf {E}}_{Z}, \end{aligned}$$

where

8 $$\begin{aligned} \lambda _1^{(3)}=\frac{R}{r k \lambda _z^{(3)}}, \quad \lambda _2^{(3)}= \frac{k r}{R}. \end{aligned}$$

It follows that the invariant $I_1$ (with the superscript $^{(3)}$ omitted temporarily for simplicity) is given by

9 $$\begin{aligned} I_1=\lambda _1^2 +\lambda _2^2+\lambda _z^2. \end{aligned}$$

The components of the Cauchy stress tensor in ${\mathscr {B}}_3$ are then

10 $$\begin{aligned} \sigma _{rr}= & {} -p+2\frac{\partial \Psi }{\partial I_1} \lambda _1^2, \end{aligned}$$

11 $$\begin{aligned} \sigma _{\theta \theta }= & {} -p+2\frac{\partial \Psi }{\partial I_1} \lambda _2^2, \end{aligned}$$

12 $$\begin{aligned} \sigma _{z z}= & {} -p+2\frac{\partial \Psi }{\partial I_1} \lambda _z^2, \end{aligned}$$

where *p* is the Lagrangian multiplier. In the absence of body forces the stress components $\sigma _{rr}$ and $\sigma _{\theta \theta }$ in ${\mathscr {B}}_3$ satisfy the equilibrium equation $\text{ div }\varvec{\sigma }={\mathbf {0}}$, which, for the considered deformation, reduces to

13 $$\begin{aligned} \frac{\mathrm {d} \sigma _{rr}}{\mathrm {d} r} + \frac{\sigma _{rr}-\sigma _{\theta \theta }}{r} = 0, \end{aligned}$$

and the associated zero-traction boundary conditions are $\sigma _{rr}=0$ for $r=r^{(\mathrm {i})}, r^{(\mathrm {o})}$.

On integration and use of the latter boundary conditions Eq. (13) gives

14 $$\begin{aligned} \int ^{r^{(\mathrm {o})}}_{r^{(\mathrm {i})}} \frac{\sigma _{\theta \theta }-\sigma _{rr}}{r} \mathrm {d} r =0, \end{aligned}$$

which, on substitution from (10) and (11, 12), can be used to obtain $r^{(\mathrm {i})}$ in ${\mathscr {B}}_3$ (and $r^{(\mathrm {o})}$ from (5)) when the initial radii $R^{(\mathrm {i})}$ and $R^{(\mathrm {o})}$ and *k* and $\gamma$ are known. Hence, given $\Psi$, all the Cauchy stress components can be obtained explicitly in ${\mathscr {B}}_3$.

It should be emphasized that $\varvec{\sigma }$ is not strictly a residual stress since the presence of the components $\sigma _{zz}$ requires appropriate non-zero boundary conditions, whereas true residual stress is associated with zero-traction boundary conditions. However, the axial force *N* required to maintain the circular cylindrical configuration ${\mathscr {B}}_3$^10^

15 $$\begin{aligned} N= 2 \pi \int ^{r^{(\mathrm {o})}}_{r^{(\mathrm {i})}} \sigma _{zz} r \mathrm {d} r= \pi \int ^{r^{(\mathrm {o})}}_{r^{(\mathrm {i})}} (2\sigma _{zz}-\sigma _{rr}-\sigma _{\theta \theta }) r \mathrm {d}r, \end{aligned}$$

is non-zero but small. Following, Ref. 12 we adjust the axial stress so that $\sigma _{zz}-\frac{N}{\pi [(r^{(o)})^2-(r^{(i)})^2]}$ is the approximate measure of the residual axial stress. The adjusted residual axial stress has its mean removed so that the resulting axial load vanishes.

### Two Cuts: Radial and Circumferential

**Figure 3:**
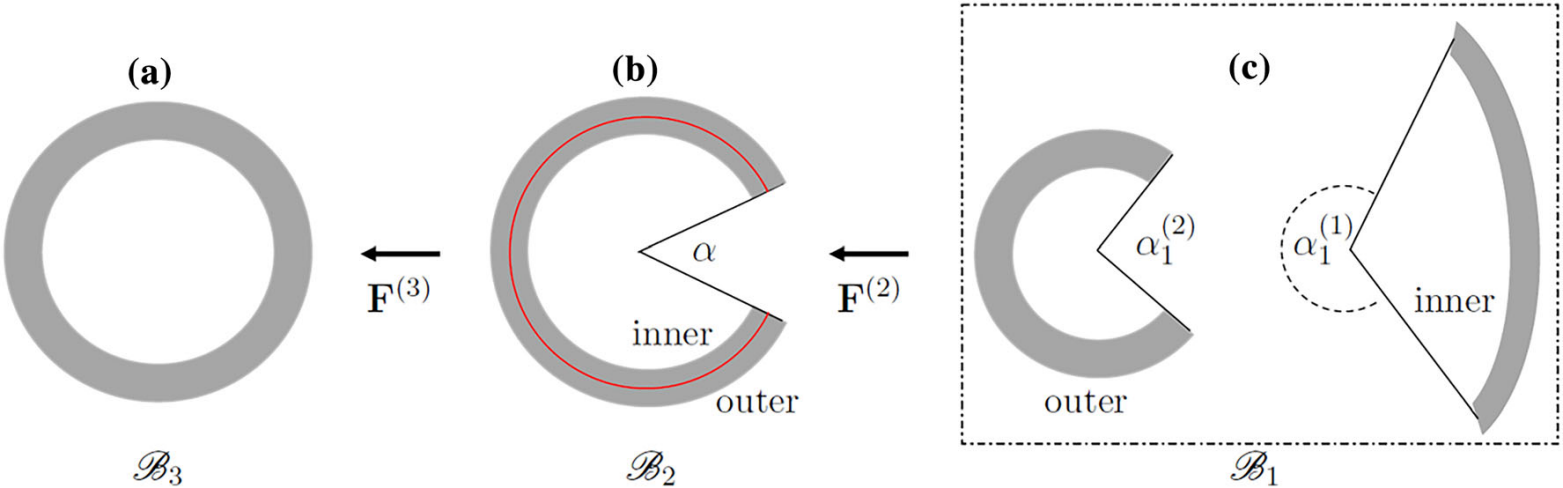
The two-cut model **a** cylindrical model of the LV as the intact ring in ${\mathscr {B}}_3$, **b** after a radial cut to ${\mathscr {B}}_2$, and **c** followed by a circumferential cut to ${\mathscr {B}}_1$. Notice that the inner sector in ${\mathscr {B}}_1$ has a negative curvature, as in Ref. 21. The red curve, at the mid-wall radius ${\bar{R}}=(R^{(\mathrm {i})}+R^{(\mathrm {o})})/2$ in (**b**), separates the inner and outer sectors which become the separate inner and outer sectrors in ${\mathscr {B}}_1$ after the circumferential cut. Appropriate axial and torsional loads are required to maintain the shapes in ${\mathscr {B}}_2$ and ${\mathscr {B}}_3.$

For the two-cut model we consider the separation of the sector in ${\mathscr {B}}_2$ into two separate circular cylindrical sectors (inner and outer) by means of a circumferential cut around the mid-wall in ${\mathscr {B}}_2$ at radius ${\bar{R}}=(R^{(\mathrm {i})}+R^{(\mathrm {o})})/2$. The two new sectors form the configuration ${\mathscr {B}}_1$, which is now taken as the stress-free reference configuration. Thus, ${\mathscr {B}}_2$ is no longer stress free but requires torsional and axial loads to maintain its circular cylindrical shape. It is, however, residually stressed in the sense that there is no traction on its curved surfaces. The transition from ${\mathscr {B}}_2$ to ${\mathscr {B}}_3$ is now different from that in the one-cut model. The stress in ${\mathscr {B}}_3$ is calculated from the constitutive laws based on the reference configuration ${\mathscr {B}}_1$ with the appropriate deformation gradients from the two sectors in ${\mathscr {B}}_1$ to ${\mathscr {B}}_3$. The transition from ${\mathscr {B}}_1$ to ${\mathscr {B}}_2$ to ${\mathscr {B}}_3$ is depicted in Fig. 3.

We note, in particular, that the inner sector in ${\mathscr {B}}_1$ has a negative curvature. The geometry in ${\mathscr {B}}_1$ is described in terms of cylindrical polar coordinates $\{R_j, \Theta_j, Z_j \}$, with subscripts *j* = 1 and 2 corresponding to the inner and outer sectors, respectively. Thus,

16 $$\begin{aligned}&R_{1}^{(\mathrm {i})} \leqslant R_1 \leqslant R_{1}^{(\mathrm {o})}, \quad \nonumber \\&\quad - \pi + \frac{\alpha ^{(1)}_1}{2}\leqslant \Theta _1\leqslant \pi -\frac{\alpha ^{(1)}_1}{2}, \quad 0\leqslant Z_1 \leqslant L_1 , \end{aligned}$$

17 $$\begin{aligned}&R_{2}^{(\mathrm {i})} \leqslant R_2 \leqslant R_{2}^{(\mathrm {o})}, \quad \nonumber \\&\quad \frac{\alpha ^{(2)}_1}{2}\leqslant \Theta _2\leqslant 2 \pi - \frac{\alpha ^{(2)}_1}{2}, \quad 0\leqslant Z_2 \leqslant L_1 , \end{aligned}$$

where $R_{j}^{(\mathrm {i})}$, $R_{j}^{(\mathrm {o})}$, $\alpha ^{(j)}_1$, $j=1,2$, and *L*_1_ denote the inner and outer radii, the opening angles, and the tube length in ${\mathscr {B}}_1$. In ${\mathscr {B}}_2$, $R_{1}^{(\mathrm {i})}$ and $R_{2}^{(\mathrm {i})}$ both become ${\bar{R}}$, while $R_{1}^{(\mathrm {o})}$ and $R_{2}^{(\mathrm {o})}$ translate to $R^{(\mathrm {i})}$ and $R^{(\mathrm {o})}$, respectively, the opening angle is $\alpha$ and the axial length *L*.

For each sector, the isochoric deformation from ${\mathscr {B}}_1$ to ${\mathscr {B}}_2$ can be written as

18 $$\begin{aligned} {\mathbf {X}}=R {\mathbf {E}}_R+Z{\mathbf {E}}_Z, \end{aligned}$$

with

19 $$\begin{aligned} R= & {} \sqrt{\frac{{R^{(\mathrm {i})}_1}^{2}-R_1^{2}}{k_1\lambda _Z^{(21)}}+{\bar{R}}^2},\nonumber \\ \Theta= & {} \pi -k_1\Theta _1,\quad Z=\lambda _Z^{(21)}Z_1 \end{aligned}$$

20 $$\begin{aligned} R= & {} \sqrt{\frac{R_2^{2}-{R^{(\mathrm {i})}_2}^{2}}{k_2\lambda _Z^{(22)}}+{\bar{R}}^2},\quad \nonumber \\ \Theta= & {} k_2(\Theta _2-\pi )+\pi ,\quad Z=\lambda _Z^{(22)}Z_2 \end{aligned}$$

for the inner and outer sectors, respectively, where $k_j=(2\pi -\alpha)/(2\pi -\alpha ^{(j)}_1), j=1,2$ , and $\lambda^{2j}$ is the axial stretch of sector *j* = 1 and 2 in ${\mathscr {B}}_1$ respectively. Note that the negative curvature of the inner sector in ${\mathscr {B}}_1$ depicted in Fig. 3 is captured by the expression for *R* in (19), and that structural compatibility in ${\mathscr {B}}_2$ is ensured since the two expressions for *R* match at ${\bar{R}}$.

As indicated in Fig. 3 the deformation gradient from ${\mathscr {B}}_1$ to ${\mathscr {B}}_2$ is denoted ${\mathbf {F}}^{(2)}$, which is shorthand notation for the two separate deformation gradients from the two sectors in ${\mathscr {B}}_1$ to ${\mathscr {B}}_2$. These are denoted ${\mathbf {F}}^{(2j)},\,j=1,2$, and given by

21 $$\begin{aligned} {\mathbf {F}}^{(2j)} =\lambda _1^{(2j)} {\mathbf {E}}_{R_j}\otimes {\mathbf {E}}_{R} +\lambda _2^{(2j)}{\mathbf {E}}_{\Theta_j }\otimes {\mathbf {E}}_{\Theta } + \lambda _Z^{(2j)} {\mathbf {E}}_{Z_j} \otimes {\mathbf {E}}_{Z}, \end{aligned}$$

where

22 $$\begin{aligned} \lambda _1^{(2j)}= & {} \frac{R_j}{ k_jR \lambda _Z^{(2j)}}, \quad \lambda _2^{(2j)}= \frac{k_j R}{R_j},\quad \nonumber \\ \lambda _Z^{(2j)}= & {} {\lambda _1^{(2j)}}^{-1}{\lambda _2^{(2j)}}^{-1}, \quad j=1,2. \end{aligned}$$

Similarly to the one-cut model, the equilibrium equation in ${\mathscr {B}}_2$ yields

23 $$\begin{aligned}&\frac{\mathrm {d} \sigma _{RR}}{\mathrm {d} R} + \frac{\sigma _{RR}-\sigma _{\Theta \Theta }}{R} = 0,\quad \nonumber \\&\quad \int ^{R^{(\mathrm {o})}}_{R^{(\mathrm {i})}} \frac{\sigma _{\Theta \Theta }-\sigma _{RR}}{R} \mathrm {d} R =0. \end{aligned}$$

Equation (23)$_1$ can be rearranged as

24 $$\begin{aligned} \sigma _{\Theta \Theta } = \frac{\mathrm {d}}{\mathrm {d} R}( R\sigma _{RR}), \end{aligned}$$

from which it follows, on use of the zero-traction boundary conditions $\sigma _{RR}=0$ on $R^{(\mathrm {i})}$ and $R^{(\mathrm {o})}$, that

25 $$\begin{aligned} \int ^{R^{(\mathrm {o})}}_{R^{(\mathrm {i})}} \sigma _{\Theta \Theta } \mathrm {d} R =0, \end{aligned}$$

i.e. the mean value of $\sigma _{\Theta \Theta }$ through the thickness is zero.

It is assumed that is there is no bending moment on the faces $\Theta =\alpha/2$ and $\Theta =2\pi -\alpha/2$ in ${\mathscr {B}}_2$, which yields

26 $$\begin{aligned} \int ^{R^{(\mathrm {o})}}_{R^{(\mathrm {i})}} \sigma _{\Theta \Theta } R\mathrm {d} R=0 . \end{aligned}$$

Substitution of $\sigma _{\Theta \Theta }$ from (24) into this equation followed by integration by parts and a further application of the zero-traction boundary conditions leads to

27 $$\begin{aligned} \int ^{R^{(\mathrm {o})}}_{R^{(\mathrm {i})}} \sigma _{RR} R\mathrm {d} R=0. \end{aligned}$$

Eqs. (23)$_2$, (26) and (27) are solved with Eqs. (19) and (20) to obtain the radii $R^{(\mathrm {i})}$ and $R^{(\mathrm {o})}$, and the angle $\alpha$ of the sector in ${\mathscr {B}}_2$. The deformation gradient associated with the transition ${\mathbf {F}}_{1\rightarrow 3}$ from ${\mathscr {B}}_1$ to ${\mathscr {B}}_3$ has the form ${\mathbf {F}}^{(3)} {\mathbf {F}}^{(2)}$, where ${\mathbf {F}}^{(3)}$ is given by Eq. (7) from the one-cut approach, and ${\mathbf {F}}^{(2)}$ is either ${\mathbf {F}}^{(21)}$ or ${\mathbf {F}}^{(22)}$, as given in Eq. (21). Note that ${\mathbf {F}}^{(3)} {\mathbf {F}}^{(21)}$ and ${\mathbf {F}}^{(3)} {\mathbf {F}}^{(22)}$ are the values obtained for the inner and outer sectors, respectively, and these must match at the interface ${\bar{R}}$, i.e. the deformation gradient must be continuous in ${\mathscr {B}}_2$, which implies that $k_1/R_1^{(\mathrm {i})}=k_2/R_2^{(\mathrm {i})}$ at the interface. Enforcing of this requirement will ensure that when traction continuity is applied *p* is continuous and hence that all the stress components are continuous, in particular that $\sigma _{\Theta \Theta }$ is continuous.

Hence, for the two-cut model, we require geometric information, e.g. $R^{(i)}_1$, $R^{(i)}_2$, $\alpha^{(1)} _1$, $\alpha^{(2)} _2$ in ${\mathscr {B}}_1$, from experiments (Fig. 1c). Then the geometric information of ${\mathscr {B}}_2$ is obtained from the two-cut model. The residual stress of the intact-ring configuration, ${\mathscr {B}}_3$, can now be solved using the same equilibrium equations as (23), (26) and (27), except the deformation gradient is now ${\mathbf {F}}^{(3)} {\mathbf {F}}^{(2)}$.

An expression for the required axial load *N* for the intact ring is obtained from a formula similar to that in (15), and the corresponding residual axial stress is adjusted to make the resulting axial load vanish.

### Four cuts: one radial and three circumferential

**Figure 4:**
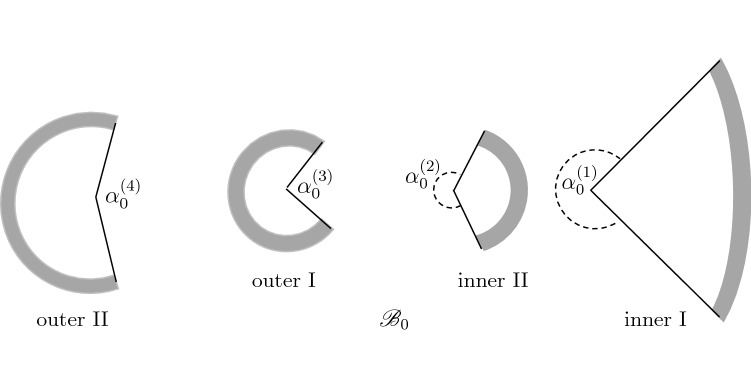
The stress-free configuration ${\mathscr {B}}_0$ consisting of the four sectors obtained by circumferential cuts of the two sectors in ${\mathscr {B}}_1.$

The effect of two further circumferential cuts, one in each of the two separated sectors, is now considered in order to assess if there is any significant change in the resulting calculated residual stress compared with that obtained with a single circumferential cut, although this is not a test that has been carried out experimentally. For definiteness we consider taking a circumferential cut along the mid-wall of each of the two sectors of the two-cut model, leading to the four separate sectors depicted in Fig. 4. In this model we assume that the resulting configuration ${\mathscr {B}}_0$ is stress-free with no further reversal of the curvature, so that the two sectors in ${\mathscr {B}}_1$ are no longer stress free.

Each of the four sectors in ${\mathscr {B}}_0$ is described in terms of cylindrical polar coordinates $\{\rho ,\phi ,\zeta \}$ according to

28 $$\begin{aligned}&\rho _1^{(\mathrm {i})} \leqslant \rho \leqslant \rho _1^{(\mathrm {o})}, \quad -\pi + \frac{\alpha ^{(1)}_0}{2} \leqslant \phi \leqslant \pi -\frac{\alpha ^{(1)}_0}{2}, \nonumber \\&\quad 0\leqslant \zeta \leqslant L_0 \quad \mathrm {(inner}\ I), \end{aligned}$$

29 $$\begin{aligned}&\rho _2^{(\mathrm {i})} \leqslant \rho \leqslant \rho _2^{(\mathrm {o})}, \quad -\pi + \frac{\alpha ^{(2)}_0}{2} \leqslant \phi \leqslant \pi -\frac{\alpha ^{(2)}_0}{2}, \nonumber \\&\quad 0\leqslant \zeta \leqslant L_0\quad \mathrm {(inner}\ II), \end{aligned}$$

30 $$\begin{aligned}&\rho _3^{(\mathrm {i})} \leqslant \rho \leqslant \rho _3^{(\mathrm {o})}, \quad \frac{\alpha ^{(3)}_0}{2}\leqslant \phi \leqslant 2 \pi - \frac{\alpha ^{(3)}_0}{2} , \nonumber \\&\quad 0\leqslant \zeta \leqslant L_0 \quad \mathrm {(outer}\ I), \end{aligned}$$

31 $$\begin{aligned}&\rho _4^{(\mathrm {i})} \leqslant \rho \leqslant \rho _4^{(\mathrm {o})}, \quad \frac{\alpha ^{(4)}_0}{2}\leqslant \phi \leqslant 2 \pi - \frac{\alpha ^{(4)}_0}{2}, \nonumber \\&\quad 0\leqslant \zeta \leqslant L_0 \quad \mathrm {(outer}\ II), \end{aligned}$$

where $\rho _n^{(\mathrm {i})}$, $\rho _n^{(\mathrm {o})}$, $\alpha ^{(n)}_0$, $n=1,2,3,4$, and *L*_0_ are the internal radii, the external radii, opening angles, and the lengths of the four sectors in ${\mathscr {B}}_0$, and the notations *I* and *II* are identified in Fig. 4.

In ${\mathscr {B}}_1$, the geometries of the two sectors are described in terms of polar coordinates $\{R_j, \Theta_j , Z_j\}$, with indices 1 and 2, as in Eqs. (16) and (17). Next, in ${\mathscr {B}}_2$, $R^{(\mathrm {i})}$, $R^{(\mathrm {o})}$, $\alpha$, *L* denote the internal and external radii, the opening angle and the length of the single sector according to (19)–(20).

The deformations from the four sectors in ${\mathscr {B}}_0$ to the two sectors in ${\mathscr {B}}_1$ are described by

32 $$\begin{aligned} R_1= & {} \sqrt{\frac{\rho ^2-{\rho _1^{(\mathrm {i})}}^2}{k_{11}\lambda _Z^{(11)}}+{\bar{R}}_1^2},\quad \Theta _1=k_{11}\phi ,\quad \nonumber \\ Z_1= & {} \lambda _Z^{(11)}\zeta ,\quad \text{(inner } \text{ I) }, \end{aligned}$$

33 $$\begin{aligned} R_1= & {} \sqrt{\frac{\rho ^2-{\rho _2^{(\mathrm {o})}}^2}{k_{12}\lambda _Z^{(12)}}+{\bar{R}}_1^2},\quad \Theta _1=k_{12}\phi ,\quad \nonumber \\ Z_1= & {} \lambda_Z^{(12)}\zeta ,\quad \text{(inner } \text{ II) }, \end{aligned}$$

34 $$\begin{aligned} R_2= & {} \sqrt{\frac{\rho ^2-{\rho _3^{(\mathrm {o})}}^2}{k_{23}\lambda _Z^{(23)}}+{\bar{R}}_2^2},\quad \Theta _2=k_{23}(\phi -\pi )+\pi ,\quad \nonumber \\ Z_2= & {} \lambda _Z^{(23)}\zeta ,\quad \text{(outer } \text{ I) }, \end{aligned}$$

35 $$\begin{aligned} R_2= & {} \sqrt{\frac{\rho ^2-{\rho _4^{(\mathrm {i})}}^2}{k_{24}\lambda _Z^{(24)}}+{\bar{R}}_2^2},\quad \nonumber \\ \Theta _2= & {} k_{24}(\phi -\pi )+\pi ,\quad Z_2=\lambda _Z^{(24)}\zeta ,\quad \text{(outer } \text{ II) }, \end{aligned}$$

where

$$\begin{aligned} k_{1n}= & {} (2\pi -\alpha _1^{(1)})/(2\pi -\alpha _0^{(n)}),\quad n=1,2,\quad \\ k_{2n}= & {} (2\pi -\alpha _1^{(2)})/(2\pi -\alpha _0^{(n)}),\quad n=3,4,\\ \lambda _1^{(1n)}= & {} \frac{R_1}{\rho k_{1n} \lambda _Z^{(1n)}},\quad \\ \lambda _2^{(1n)}= & {} \frac{k_{1n}\rho }{R_1}, \quad \\ \lambda _Z^{(1n)}= & {} {\lambda _1^{(1n)}}^{-1}{\lambda _2^{(1n)}}^{-1},\quad n=1,2,\\ \lambda _1^{(2n)}= & {} \frac{R_2}{\rho k_{2n} \lambda _Z^{(2n)}},\quad \\ \lambda _2^{(2n)}= & {} \frac{k_{2n} \rho }{R_2}, \quad \\ \lambda _Z^{(2n)}= & {} {\lambda _1^{(2n)}}^{-1}{\lambda _2^{(2n)}}^{-1},\quad n=3,4, \end{aligned}$$

and

$$\begin{aligned} {\bar{R}}_j=\frac{1}{2}(R_j^{(\mathrm {i})}+R_j^{(\mathrm {o})}),\quad j=1,2. \end{aligned}$$

The deformation gradients from ${\mathscr {B}}_0$ in Fig. 4 to ${\mathscr {B}}_1$ in Fig. 3 are

36 $$\begin{aligned} {\mathbf {F}}^{(1n)}= & {} \lambda _{1}^{(1n)}{\mathbf {E}}_{R_1}\otimes {\mathbf {e}}_{\rho }+\lambda _{2}^{(1n)} {\mathbf {E}}_{\Theta_1 } \otimes {\mathbf {e}}_{\phi }+ \lambda _{Z}^{(1n)} {\mathbf {E}}_{Z_1}\otimes {\mathbf {e}}_\zeta ,\quad n=1,2, \end{aligned}$$

37 $$\begin{aligned} {\mathbf {F}}^{(2n)}= & {} \lambda _{1}^{(2n)}{\mathbf {E}}_{R_2}\otimes {\mathbf {e}}_{\rho } +\lambda _{2}^{(2n)} {\mathbf {E}}_{\Theta_2 }\nonumber\otimes {\mathbf {e}}_{\phi }+ \lambda _{Z}^{(2n)} {\mathbf {E}}_{Z_2}\otimes {\mathbf {e}}_\zeta ,\quad n=3,4. \end{aligned}$$

In this model there are four separate deformation gradients from ${\mathscr {B}}_0$ to ${\mathscr {B}}_1$, pairs of which have to be continuous in ${\mathscr {B}}_1$. Then, the two separate deformation gradients from ${\mathscr {B}}_1$ to ${\mathscr {B}}_2$ also have to be continuous, and the deformation gradient from ${\mathscr {B}}_2$ to ${\mathscr {B}}_3$ likewise has to be continuous. The transformations between the various internal and external radii are listed in Table 1.

**Table 1:** Transformations between the various internal and external radii in the different configurations.

${\mathscr {B}}_0$		${\mathscr {B}}_1$		${\mathscr {B}}_2$		${\mathscr {B}}_3$
$\rho _1^{(\mathrm {i})}$	$\rightarrow$	${\bar{R}}_1$				
$\rho _1^{(\mathrm {o})}$	$\rightarrow$	${R}_1^{(\mathrm {o})}$	$\rightarrow$	$R^{(\mathrm {i})}$	$\rightarrow$	$r^{(\mathrm {i})}$
$\rho _2^{(\mathrm {i})}$	$\rightarrow$	${R}_1^{(\mathrm {i})}$	$\rightarrow$	$\frac{1}{2}(R^{(\mathrm {i})}+R^{(\mathrm {o})})$		
$\rho _2^{(\mathrm {o})}$	$\rightarrow$	${\bar{R}}_1$				
$\rho _3^{(\mathrm {i})}$	$\rightarrow$	${R}_2^{(\mathrm {i})}$	$\rightarrow$	$\frac{1}{2}(R^{(\mathrm {i})}+R^{(\mathrm {o})})$		
$\rho _3^{(\mathrm {o})}$	$\rightarrow$	${\bar{R}}_2$				
$\rho _4^{(\mathrm {i})}$	$\rightarrow$	${\bar{R}}_2$				
$\rho _4^{(\mathrm {o})}$	$\rightarrow$	${R}_2^{(\mathrm {o})}$	$\rightarrow$	$R^{(\mathrm {o})}$	$\rightarrow$	$r^{(\mathrm {o})}$

In ${\mathscr {B}}_1$, the radial equilibrium equation for each of the two sectors yields

38 $$\begin{aligned} \int ^{R_{j}^{(\mathrm {o})}}_{R_j^{(\mathrm {i})}} \frac{\sigma _{\Theta_j \Theta_j }-\sigma _{R_jR_j}}{R_j} \mathrm {d} R =0, \quad j=1,2. \end{aligned}$$

The radial traction $\sigma _{R_jR_j}$ should be continuous across each interface ${\bar{R}}_j,\,j=1,2$, and hence, since the deformation gradient is required to be continuous, *p* is continuous and the other stress components are also continuous. For the considered deformation, with $\lambda _{Z_j}$ given, continuity of both the radial and circumferential stresses guarantees that both *p* and the deformation are continuous. Note that continuity of the circumferential stress at the interfaces in ${\mathscr {B}}_1$ requires that

39 $$\begin{aligned} \sigma _{\Theta_j \Theta_j }\quad \text{ is } \text{ continuous } \text{ for } \quad R_1={\bar{R}}_1\ (R_2={\bar{R}}_2)\ \text{ in }\ \text{ the } \text{ inner } \text{(outer) } \text{ sector }. \end{aligned}$$

Also, similarly to the two-cut model,

40 $$\begin{aligned} \int ^{R_j^{(\mathrm {o})}}_{R_j^{(\mathrm {i})}} \sigma _{\Theta_j \Theta_j } R_j \mathrm {d} R = \int ^{R_j^{(\mathrm {o})}}_{R_j^{(\mathrm {i})}} \sigma _{R_jR_j} R_j \mathrm {d}R=0, \quad j=1,2. \end{aligned}$$

The corresponding deformation gradient from ${\mathscr {B}}_1$ to ${\mathscr {B}}_2$ is ${\mathbf {F}}_{0 \rightarrow 2}= {\mathbf {F}}^{(2)} {\mathbf {F}}^{(1)}$, where again ${\mathbf {F}}^{(2)}$ is either ${\mathbf {F}}^{(21)}$ or ${\mathbf {F}}^{(22)}$, ${\mathbf {F}}^{(1)}$ is the appropriate ${\mathbf {F}}^{(1n)}$, $n=1,2$, or ${\mathbf {F}}^{(2n)}$, $n=3,4$, and the governing equations for the single sector in ${\mathscr {B}}_2$ are

41 $$\begin{aligned}&\int ^{R^{(\mathrm {o})}}_{R^{(\mathrm {i})}} \frac{\sigma _{\Theta \Theta }-\sigma _{RR}}{R} \mathrm {d} R=0,\quad \nonumber \\&\quad \int ^{R^{(\mathrm {o})}}_{R^{(\mathrm {i})}} \sigma _{\Theta \Theta } R \mathrm {d} R = \int ^{R^{(\mathrm {o})}}_{R^{(\mathrm {i})}} \sigma _{RR}R \mathrm {d}R=0, \end{aligned}$$

as in (23)$_2$, (26) and (27).

This model involves eight independent Eqs. (38)–(41), and eight unknown geometrical parameters in ${\mathscr {B}}_0$: $\rho ^{(\mathrm {i})}_n, \alpha _0^{(n)}$, $n=1, 2, 3, 4$. The required external axial load is then calculated similar to (15), and made to vannish by adjuing the residual axial stress.

In summary, for the four-cut model, we require geometric information of both ${\mathscr {B}}_1$ and ${\mathscr {B}}_2$ from experiments. Once we obtain all the details, e.g, opening angles and radii of all the sectors in ${\mathscr {B}}_0$, we estimate the (residual) stress components $\sigma _{rr}$ and $\sigma _{\theta \theta }$ in the intact-ring configuration ${\mathscr {B}}_3$, with the total deformation gradient ${\mathbf {F}}_{0\rightarrow 3}={\mathbf {F}}^{(3)} {\mathbf {F}}^{(2)} {\mathbf {F}}^{(1)}$.

## Results

### Modelling Parameters

The reference configuration is different for the different models, so we need to define the parameters according to each specific model considered. From $${\mathscr {B}}_2$$ in the one-cut model, we assume that the axial stretch has the constant value $$\lambda _z^{(3)}=1.14$$, and $$R^{(\mathrm {i})}=2.06$$, $$R^{(\mathrm {o})}=3.20$$$$\alpha=65^\circ$$ are estimated from the experiments.^21^

For the two-cut model, in addition to the parameters used in the one-cut model, we use additional measurements in ${\mathscr {B}}_1$ of the two-cut model in Ref. 21: $R_1^{(\mathrm {i})}=5.28$, $R_{2}^{(\mathrm {i})}=1.97$, $\alpha _1^{(1)}=268^\circ$ and $\alpha _1^{(2)}=180^\circ$. We also assume that there is no axial stretch in the transformation from ${\mathscr {B}}_1$ to ${\mathscr {B}}_2$, i.e. $\lambda _{Z}^{(2j)}=1$, *j*=1,2.

For the four-cut model, in addition to the parameters used in the two-cut model, we need more geometrical information in ${\mathscr {B}}_1$, which is again estimated from the measurements^21^ as listed in Table 2. We also assume that $\lambda _{Z}^{(1n)} =\lambda _{Z}^{(2n)}=1$ for the deformation from ${\mathscr {B}}_0$ to ${\mathscr {B}}_1$, i.e. no axial deformation occurs as a result of the circumferential cuts.

**Table 2:** Measured geometrical input for the four-cut model, estimated from Ref. 21.

Configuration ${\mathscr {B}}_1$	Configuration ${\mathscr {B}}_2$
${R}_1^{(\mathrm {i})}=5.28$	$\frac{1}{2}({R}^{(\mathrm {i})}+{R}^{(\mathrm {o})})$
${R}_1^{(\mathrm {o})} =6.29$	${R}^{(\mathrm {i})}=2.06$
$\alpha _1^{(1)}=268^\circ$	$\alpha=65^\circ$
${R}_2^{(\mathrm {i})}=1.97$	$\frac{1}{2}({R}^{(\mathrm {i})}+{R}^{(\mathrm {o})})$
${R}_2^{(\mathrm {o})}=3.04$	${R}^{(\mathrm {o})}=3.20$
$\alpha _1^{(2)}=180^\circ$	$\alpha=65^\circ$

Initially, we consider a homogeneous myocardium model, for which the material parameters of the constitutive law (1) are fitted to the data of mice.^20^ This gives $a=2.21$kPa, and $b=1.8$.

However, the dramatic difference in the maximum hoop stress between the single-cut and multiple-cut models raises a question about the rationale of considering homogeneous material properties. Novak *et al*.^18^ showed that canine myocardium is heterogeneous with location dependent material properties. In particular, they showed that the mid myocardium is softer than epicardium or endocardium, although they didn’t find differences in regional (anterior wall vs septum) stiffness. We therefore also consider an inhomogeneous myocardium model, and fit the data from^18^ with the strain-energy function (1).

**Figure 5:**
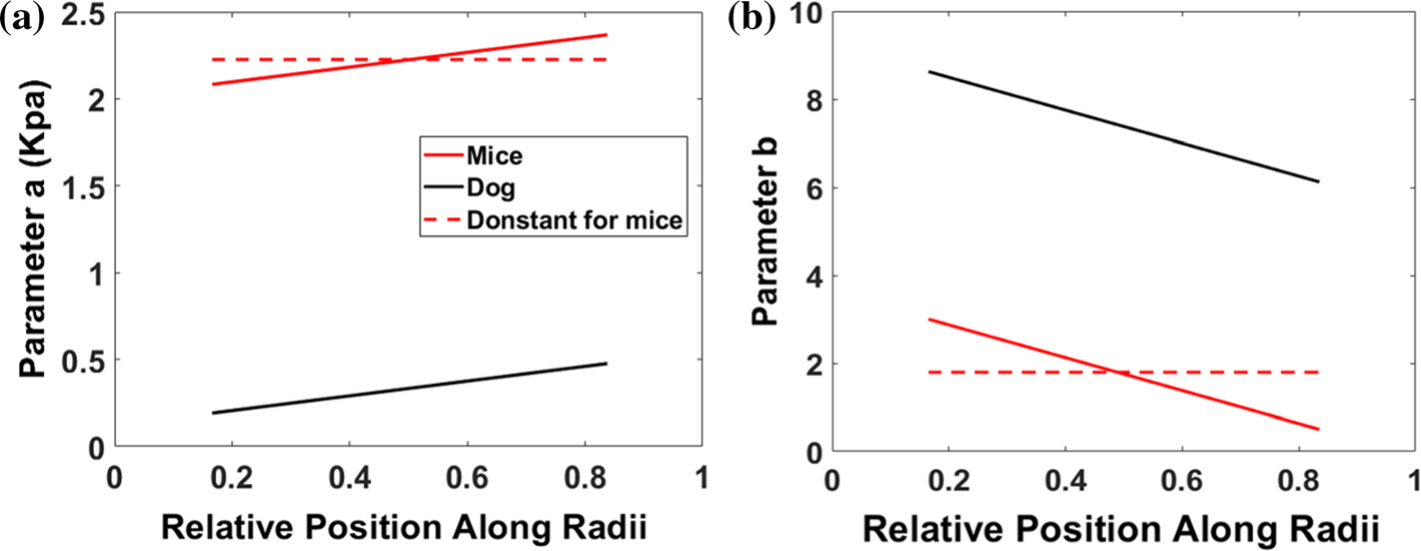
The heterogeneous material parameters *a* (left), and *b* (right), fitted to experiments. The red dashed lines indicate the constants used for the homogeneous models, and the red solid lines are for the heterogeneous mice models, with the spatial distributions taken from the canine data in Ref. 18 (black solid lines).

The parameters are spatially dependent, as shown in Fig. 5. Since our model is for mice, and there are no experimental data on the heterogeneous properties of mice myocardium, we take the spatial variation of the canine data, but keep the mean values of the mice data from our fitted parameters, and include these in our calculations.

### Results for the Homogeneous Myocardium Model

The geometry of the intact ring predicted in the three different models is summarized in Table 3, and compared with the measured data in Ref. 21.

It is clear that the agreement of the estimated radius and thickness of the unloaded configuration gets better as the number of cuts increases. Although in principle the true zero-stress configuration requires infinite cuts, Table 3 suggests that two or four cuts provides a good approximation of the zero-stressed configuration, given that the measured geometry is not exactly circular, although it is assumed to be circular in each model.

**Table 3:** Computed intact ring for the homogeneous models, compared to measurements.^21^

Measurements (mm)	One-cut model	Two-cut model	Four-cut model
$r^{(\mathrm {i})}=0.565$	0.555	0.347	0.364
$r^{(\mathrm {o})}=2.185$	2.147	1.9561	1.9950
$r^{(\mathrm {o})}-r^{(\mathrm {i})}=1.620$	1.5920	1.6438	1.6310
Difference in thickness	1.73%	1.47%	0.68%

**Figure 6:**
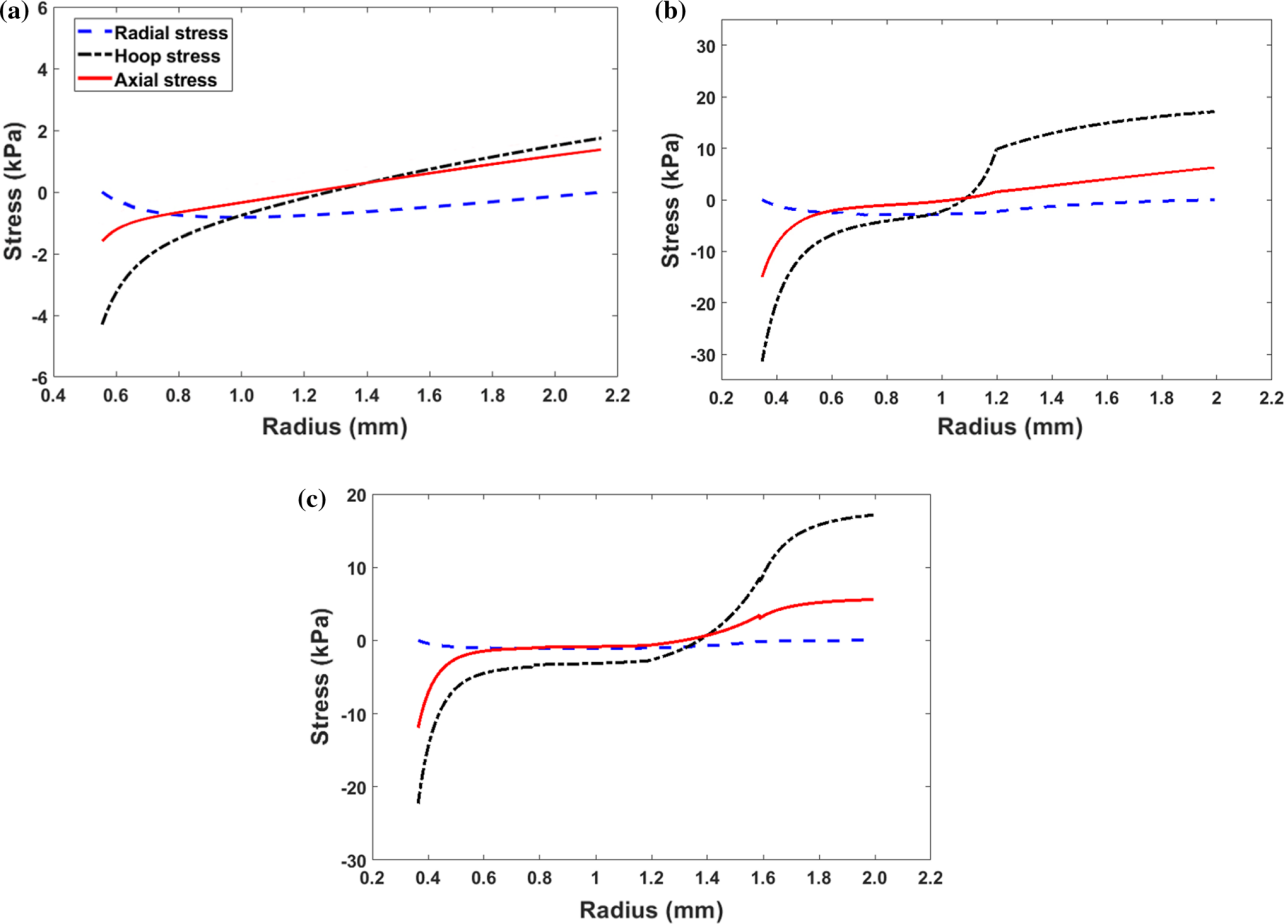
Distribution of the residual stress components in the intact ring from **a** single cut, **b** two-cut, and **c** four-cut models based on the homogenous material assumption.

The components of the residual stress distribution in the intact-ring configuration ${\mathscr {B}}_3$ from the three different models are shown in Fig. 6. The residual axial stress for each model is adjusted to remove the impact of the non-zero values of N (=$5.67\times 10^{-3}$, $1.71\times 10^{-3}$, and $1.42\times 10^{-3}$, respectively, in the one-cut, two-cut, and four-cut models.) Although the overall distributions are similar in all these models, there is marked difference in the magnitudes of the hoop stresses. In particular, the maximum $\sigma _{\theta \theta }$ is 1.75, 17.13, and 17.15 kPa, respectively, for the one-cut, two-cut, and four-cut models. Comparing to the one-cut model, the ratio of the maximum hoop stresses over the single cut is about 9.78 times for the two-cut model, and 9.80 times for the four-cut model. We also notice that although the four-cut model gives much smoother stress distribution, the two-cut model leads to a similar magnitude of the maximum hoop stress. This suggests that the significant rise in the residual stress is due to the negative curvature at the first circumferential cut.

### Results for the Heterogeneous Myocardium Model

**Figure 7:**
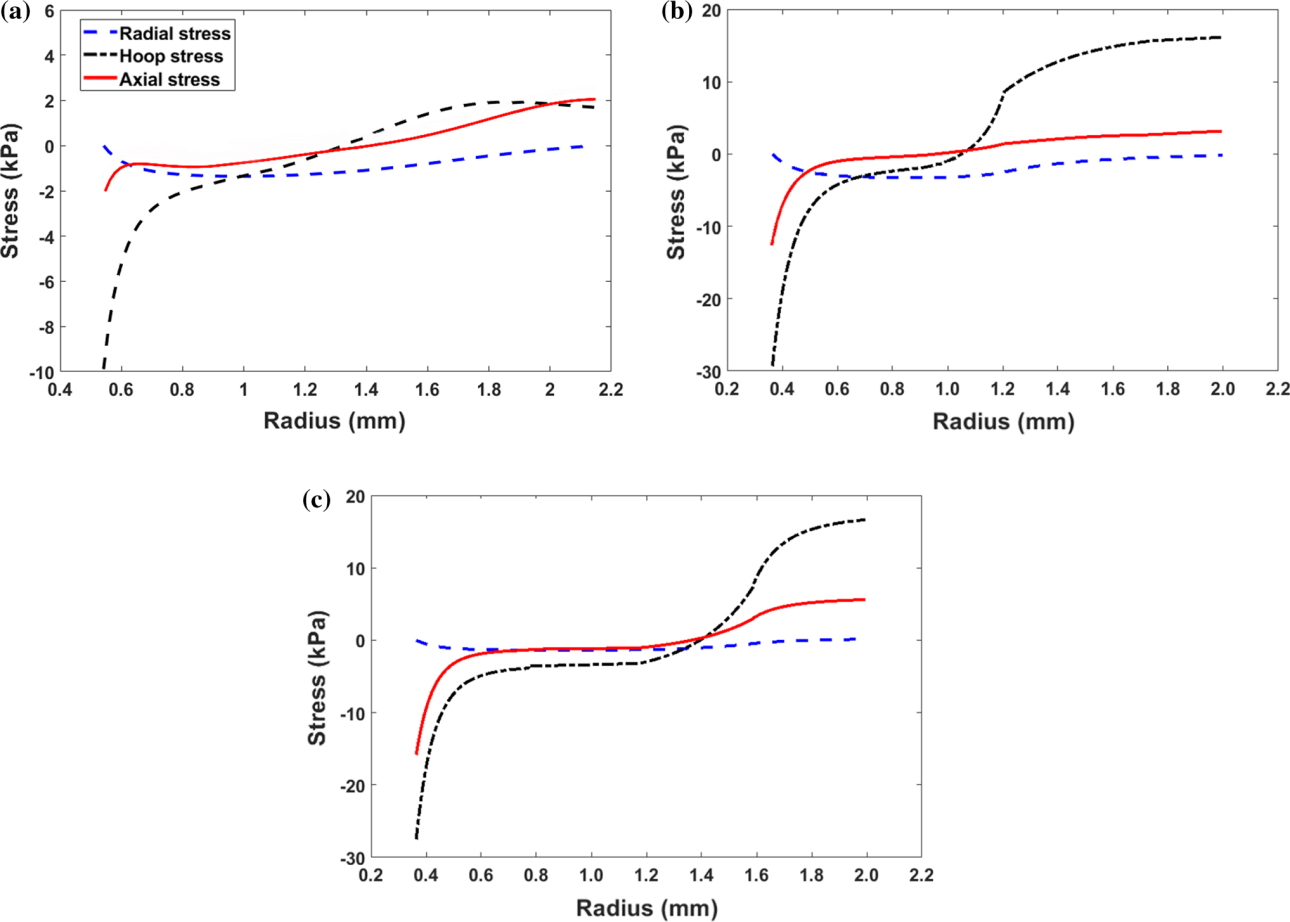
Distribution of the residual stress components from **a** one-cut, **b** two-cut, and **c** four-cut models based on the heterogeneous myocardium assumption.

The results of the intact ring from the heterogeneous myocardium model are computed. Again, the residual axial stress for each model is adjusted to remove the impact of the non-zero values of N (=$4.59\times 10^{-3}$, $1.35\times 10^{-3}$, and $1.09\times 10^{-3}$, respectively, in the one-cut, two-cut, and four-cut models.) . All the stess compoenets are plotted in Fig. 7, which shows that the maximum $\sigma _{\theta \theta }$ is 1.85, 16.12, and 16.63 kPa, respectively, for the one-cut, two-cut, and four-cut models. The ratio of the maximum hoop stresses over the single cut is slightly lower than that of the homogeneous material, at about 8.71 times for the two-cut model and 8.73 times for the four-cut model. Again, apart from smoother and somewhat different stress distributions, the four-cut model predicts very similar maximum hoop stress as the two-cut model.

## Discussion

The issue of multiple cuts has been studied before. In particular, Fung suggested that one radial cut might be sufficient to release all the residual stress. This was supported by his experiments which showed that after two or more radial cuts, no obvious deformation occurs from the one-radial-cut configuration of the arteries.^6^ Using our models we can show, however, that multiple radial cuts do not indeed release more residual stress, since due to the symmetry of the considered geometry, once a radial cut is made, no further elastic deformation can occur after more radial cuts.

In other words, the deformation gradient in each of our different models is independent of the azimuthal angle and the solutions are also independent of this angle. However, this does not indicate that the one-cut configuration is a stress-free one. To further release residual stress circumferential cuts following a radial cut are necessary. We note that even with multiple cuts, we may not release all the residual stresses. In principle, only infinite cuts can release all the residual stresses. This explains why the stress distributions are not smooth and appear to have deflections around where the cuts are, since we have to assume the configuration after two or four cuts is stress-free. This process could be improved with more cuts, though more than four cuts cannot be modelled in this paper without further experimental data. However, the curves in the four-cut model are almost smooth, the required axial load to maintain the cylindrical shape of the intact ring reduces is smaller, and the predicted intact ring thickness agrees very well with the measurements. All these tentatively suggest that the four-cut model is already a good approximation for the residual stresses. Indeed, in terms of estimating the residual stress magnitude, even the two-cut model seems to be good enough.

The fact that our two-cut and four-cut models predict a much higher (about 8-9 times!) hoop stress is perhaps not unexpected given the large negative curvature revealed by the experiments.^21^ The increased value of the residual hoop stress agrees with the rough estimation by Omens *et al*.^21^ based on a simplified concentric cylindrical shell model, where they showed that the maximum loop stress was about 20kPa, which is close to our estimation of about 17kPa.

**Figure 8:**
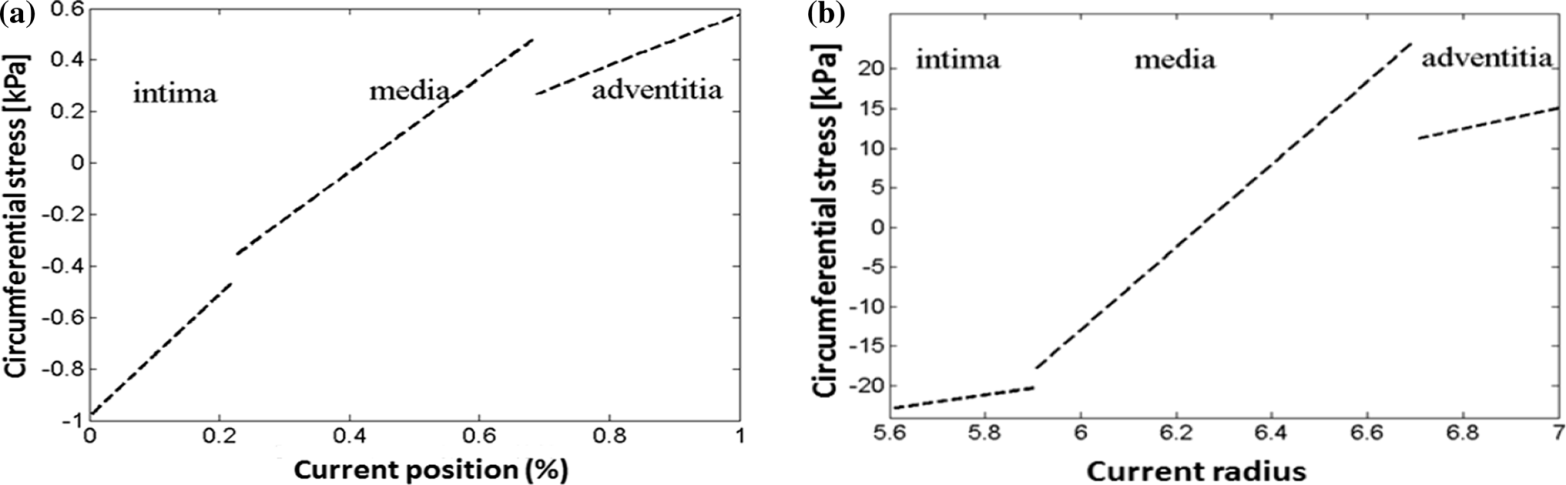
Residual stress distributions through the intima, media and adventitia of the artery wall as functions of the radial coordinate *r*: **a** residual stress is recomputed here using a one-cut model, all other parameters being the same, so that the comparison can be made with the multi-layer approach, and **b** the original result with layer separations from Ref. 12, reused with permission

We argue that the significant stress underestimate of the one-cut model does not just occur in the heart models. In the residual stress modelling of arteries, by treating the artery wall as three separate layers (intima, media and adventitia), and measuring the opening angles for each of the three layers, Holzapfel and Ogden^12^ have essentially developed a three-cut model (one radial cut followed by two circumferential cuts, in this case separating the layers with different properties). Their model is also heterogeneous, as different material parameters are used for different layers. We now compare the residual stress distribution across the artery wall in Fig. 8 using their three-cut approach and the one-cut model. The results from the one-cut (or one-layer) model are reproduced here using the same parameters as in Ref. 12. The maximum values of the hoop stress and their ratio to the one-cut model results in different layers of the three-cut model are listed in Table 4, which shows that the ratio of the hoop stress in the different layers ranges from 24 to 50 times. This difference is even greater than that of the mouse heart.

In this study, we assume that the deformation gradient is diagonal. Note this is in general not true for the heart under loading.^8^ However, since residual stresses are estimated from the unloaded configuration, the fibres are in general coiled and do not bear the load. Therefore the myocardium behaves like an isotropic material, and the radial, circumferential and axial directions are the principal directions of the deformation, i.e. the deformation gradient is diagonal in these directions. This is different from artery modelling, when the two families of fibres are assumed symmetrical about the axial direction. Hence, even when loaded with pressure, tensioned fibres do not alter the principal directions of the deformation. Therefore, the diagonal deformation gradient (e.g. Ref. 12) is assumed in arteries for a different reason.

Finally, we would like to state the limitations of the study. We have assumed that cross-sections of the heart are cylindrical, and all the cut segments retain their cylindrical configurations, each with its own opening angle, i.e. each is a circular cylindrical sector. Needless to say, this is not always true, particularly in opening angle studies of arteries, where a single cut of an arterial ring can have a non-uniform curvature.^25^ In addition, with the cylindrical assumption, we cannot model the experimental observation that opening angles are location-dependent, with higher values at heart apex.^21^ Indeed, the “true” residual stress can only be achieved through infinite cuts using the opening angle method, which is not practical. We believe the residual stress estimation, although an approximation, is a step forward to the physiological range compared with using the single cut opening angle method.

**Table 4:** Residual stress computed using a single-cut and the original (three-cut) HO model.^12^

Max. hoop stress (kPa)	One-cut model	Three-cut model^12^	Ratio of stresses
Adventitia	0.57	15.08	26.45
Media	0.47	23.74	50.51
Intima	− 0.97	− 23.20	23.91

## Conclusion

Based on experimental observations that a single radial cut does not release all residual stress, we have used multiple cuts to estimate the residual stress distributions in a mouse left ventricle model. Our results show that both radial and circumferential cuts are required to release the residual stresses in the middle wall of the left ventricle. Remarkably, using radial cuts alone leads to a significant underestimate of the residual stress, which will be around 8 to 9 times greater if estimated on the basis of combined radial and circumferential cuts. Similar findings apply to arteries based on the model in Ref. 12. We remark that the results are not significantly different using the homogeneous or heterogeneous material models. In addition, although the stress distributions are different and much smoother in the four-cut model, the two-cut model can already estimate the maximum hoop residual stress quite satisfactorily.
